# Accuracy of fit of implant-supported bars fabricated on definitive casts made by different dental stones

**DOI:** 10.4317/jced.54603

**Published:** 2018-03-01

**Authors:** Ioannis Kioleoglou, Argirios Pissiotis, Michalakis Konstantinos

**Affiliations:** 1 DDS, MS, Former postgraduate resident, Dept. of Prosthodontics, School of Dentistry, Faculty of Health Sciences, Aristotle University, Thessaloniki, Greece. ITI Scholar and Honorary Clinical Research Fellow, Department of Restorative Dentistry and Adult Oral Health, Barts and the London School of Medicine and Dentistry, Queen Mary University of London, United Kingdom; 2 DDS, MS, PhD, Professor and Chair, Division of Removable Prosthodontics, Dept. of Prosthodontics, School of Dentistry, Faculty of Health Sciences, Aristotle University, Thessaloniki, Greece; 3 DDS, MSc, PhD, FACP, Associate Professor and Clinical Director of Postgraduate Prosthodontics, Dept. of Prosthodontics, School of Dentistry, Faculty of Health Sciences, Aristotle University, Thessaloniki, Greece. Adjunct Associate Professor, Division of Postgraduate Prosthodontics, Dept. of Prosthodontics, Tufts University School of Dental Medicine, Boston, MA, USA

## Abstract

**Background:**

The purpose of this study was to evaluate the accuracy of fitting of an implant supported screw-retained bar made on definitive casts produced by 4 different dental stone products.

**Material and Methods:**

The dental stones tested were QuickRock (Protechno), FujiRock (GC), Jade Stone (Whip Mix) and Moldasynt (Heraeus). Three external hexagon implants were placed in a polyoxymethylene block. Definitive impressions were made using monophase high viscosity polyvinylsiloxane in combination with custom trays. Then, definitive models from the different types of dental stones were fabricated. Three castable cylinders with a machined non-enganging base were cast and connected with a very small quantity of PMMA to a cast bar, which was used to verify the marginal discrepancies between the abutments and the prosthetic platforms of the implants. For that purpose special software and a camera mounted on an optical microscope were used. The gap was measured by taking 10 measurements on each abutment, after the Sheffield test was applied. Twelve definitive casts were fabricated for each gypsum product and 40 measurements were performed for each cast. Mean, minimum, and maximum values were calculated. The Shapiro-Wilk test of normality was performed. Mann-Whitney test (*P*<.06) was used for the statistical analysis of the measurements.

**Results:**

The non-parametric Kruskal-Wallis test revealed a statistically significant effect of the stone factor on the marginal discrepancy for all Sheffield test combinations: 1. Abutment 2 when screw was fastened on abutment 1 (χ2=3, df=35.33, *P*<0.01), 2. Abutment 3 when the screw was fastened on abutment 1 (χ2=3, df=37.74, *P*<0.01), 3. Abutment 1 when the screw was fastened on abutment 3 (χ2=3, df=39.79, *P*<0.01), 4. Abutment 2 when the screw was fastened on abutment 3 (χ2=3, df=37.26, *P*<0.01).

**Conclusions:**

A significant correlation exists between marginal discrepancy and different dental gypsum products used for the fabrication of definitive casts for implant supported bars. The smallest marginal discrepancy was noted on implant supported bars fabricated on definitive casts made by Type III mounting stone. The biggest marginal discrepancy was noted on implant supported bars fabricated on definitive casts made by Type V dental stone. The marginal discrepancies presented on implant supported bars fabricated on definitive casts made by two types of Type IV dental stone were not significantly different.

** Key words:**Dental implant, passive fit, dental stones, marginal discrepancy.

## Introduction

Dental implants have been efficiently used for the rehabilitation of partially and completely edentulous patients, for more than thirty years. Predictable long term results have been achieved for both fixed and removable implant supported prostheses (1-8).

One of the major issues arising when more than one implants are restored is that of passive fit. The term ‘passive fit’ refers to the simultaneous sitting of all marginal points of a prosthesis on the corresponding transmucosal abutments or on the prosthetic platforms of the implants if castable cylinder abutments have been used. A requirement for a passive fit is the absence of any stress in the prosthesis/abutment/screw/implant complex, when functional or parafunctional loads are not exerted on the system. In the absence of this prerequisite technical complications may arise. These include screw loosening and/or fracture, as well as abutment, prosthesis and implant fractures (1,7,9-24). Furthermore, the biological complications that may occur include discomfort or pain to the patient and bone loss (4,7,25-36).

Several methods have been proposed in order to evaluate the passivity of the fit of an implant supported prosthesis. These include visual, tactile and radiographical methods, as well as use of disclosing agents, strain gauges, patient feedback and the Sheffield test. The latter is probably the method which is most commonly used. It refers to the complete fastening of one terminal screw and the examination of the fitting of the prosthesis on all other abutments to which the corresponding screws have not been fastened (37,38). The above methods are subjective and they rely on the clinical experience of the operator.

Most of the testing procedures focus on the gap between the prosthesis and the transmucosal abutments or between the prosthesis and the implants, if castable cylinders have been used. The acceptable marginal opening associated with the existence of a passive fit has changed over the years. In 1983, P-I Brånemark considered that 10μm was the maximum acceptable opening between the prosthesis and the transmucosal abutments (2). The 10μm limit as a maximum marginal discrepancy is also supported by Romero *et al.* and Abduo and Lyons (39,40). However, Klineberg and Murray have supported the notion that the existence of a 30μm opening in 10% of the abutment-implant interface is clinically acceptable (41). In 1992, Assif *et al.*have proposed that 26 μm is an acceptable marginal opening, while Jemt in 1991 and Yanase *et al.* in 1994 have concluded that the marginal opening should be 150μm or less (10,37,42). To date, there is no consensus regarding both the definition of ‘passive fit’ and the method which should be employed in order to evaluate the framework passivity (40,43-49).

Traditional fabrication of implant supported prostheses requires use of definitive casts made of dental stones. It has been demonstrated that the technique and the materials employed are very important for the accuracy of the definitive cast (50-54). Several materials, including epoxy resins, have been used in the past in an effort to improve the properties of the definitive casts, specifically surface hardness, resistance to abrasion, and detail reproduction (55-58). However, one of the major problems encountered was the polymerization shrinkage which led to pronounced dimensional instability (59). Usually, type IV and V dental stones are employed for the fabrication of the definitive casts (60-64). A characteristic of dental stones is their expansion during the setting process. The expansion varies depending on the type of stone used, and it ranges between 0.08% and 0.2% (65). Generally, almost 75% of the setting expansion observed in the first 24 hours occurs in the first hour (66). However, it has been demonstrated that setting expansion continues for a period of 96 hours (67,68). This setting expansion does not contribute to inaccuracies which will have as an effect the absence of a passive fit of a prosthesis onto the supporting implants (62), while others claim the opposite (69,70).

The purpose of the present study was to evaluate *in vitro* the accuracy of fitting of an implant supported screw-retained bar made on definitive casts produced by 4 different dental stone products. The null hypothesis was that the accuracy of fitting of the implant supported bar would not be affected by the different dental stone products.

## Material and Methods

Four dental stone products were included in this *in vitro* study ( Table 1). These dental stones were chosen because they are widely used in both the United States and the European Union.

**Table 1:**
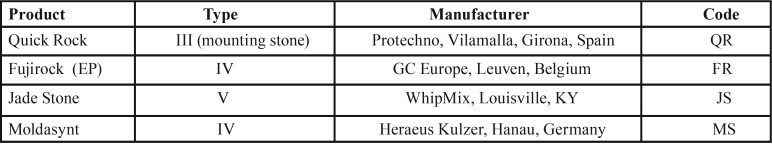
Dental Stones Studied.

**Figure 1:**
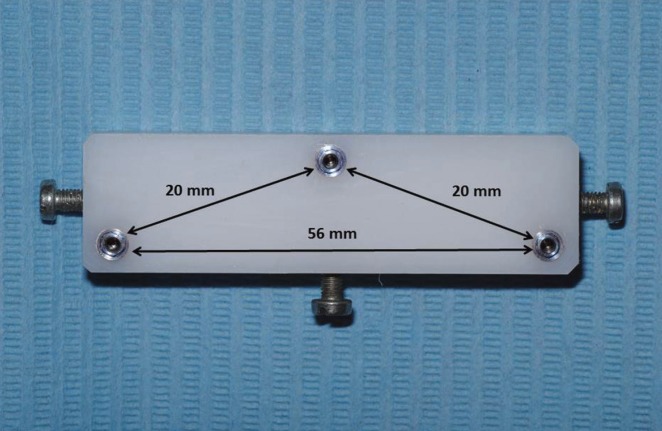
The POM block used for the purposes of this study.

**Figure 2:**
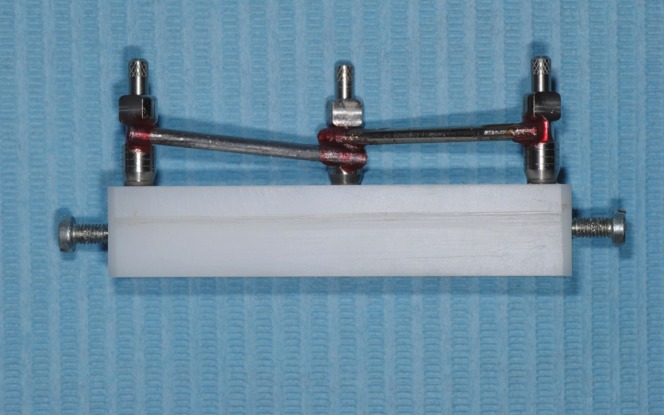
Impression copings splinted with metal rods and PMMA resin.

A 64×18×12 mm polyoxymethylene (Tecaform AH; Ensigner Inc, Washington, PA) block with a hardness of 145MPa and a modulus of elasticity of 2800MPa, was fabricated for the purposes of this study. An industrial milling machine (WMW Machinery Co) was used to drill three parallel to each other sites, with a diameter of 3.20 mm and a depth of 8 mm. Three 3.25/4.00, 8 mm long external hexagon implants (Biomet 3i) were then driven into the prepared sites with a milling machine (Ammann Girbach), so that parallelism could be ensured, and were numbered counterclockwise as 1, 2 and 3. These implants were chosen due to their very small machining tolerance (71-73). Implants’ prosthetic platforms were above the top surface of the polyoxymethylene block (74). Three screws with a length of 10 mm and a diameter of 2.75 mm were placed in 3 out of the 4 vertical surfaces of the polyoxymethylene block, in order to be used as stops during the impression procedures (Fig. 1). All included materials were from the same batch, while all testing procedures were completed by the same person (75). Before each testing procedure all joining surfaces were thoroughly cleaned with isopropyl alcohol 93o, in order to ensure an accurate fitting (76,77). One minute after the application of the alcohol the transfer impression copings (IIC12 implant EP pick-up coping; Biomet 3i) were fastened with a 10Ncm torque on the implants by means of prosthetic torque ratchet (Implant Support Systems; Lifecore Biomedical Inc.) (78,79). The accuracy of fitting between the implants and the corresponding impression copings was evaluated both visually and digitally with a 60μ tip explorer (Explorer 0701-6; ASA Dental S.p.A.) (80). Tungsten carbide rods (HM1HP, Meisinger) along with polymethylmethacrylate resin (PMMA) (Pattern Resin LS; GC America Inc) were used to connect the 3 impression copings (81). The PMMA was added in very small quantities with the bead-brush technique (82-85). Each new quantity was added after the complete polymerization of the previous one. The definitive impression was made 20 min after the placement of the last PMMA quantity, to ensure complete polymerization of the material (83), (Figs. 2).

Four identical polyvinyl chloride (PVC; Industrie Generali, S.p.A.) custom impression trays (65×20×14mm) were fabricated by using an industrial milling machine (FND 32, AVIA S.A.). The custom trays ensured a 2mm uniform thickness of impression material. Three holes corresponding to the implant positions were opened. Before the impression procedure these holes were covered with pink baseplate wax (Anutex, Toughened Dental Modelling Wax; Kemdent). The internal surfaces of the custom trays were painted with polyvinylsiloxane adhesive (Coltène Adhesive; Coltène/Whaledent AG), which was left to dry for 15 min before each impression (50,86-90). Monophase polyvinylsiloxane (Affinis Monobody/HeavyBody System360; Coltène/Whaledent AG) was used for the impression procedures (91). The material was mixed and dispensed automatically (Mixstar eMotion; DMG) through a mixing tip which was always embedded into the material in order to avoid any air entrapment (92). The material was placed with an elastomeric syringe (Penta Elastomer Syringe; 3M ESPE) around the implants and the splinted corresponding impression copings. The custom tray was then placed on the POM block with a light hand pressure until it was fully seated and kept in that position for 10 min to ensure complete polymerization of the impression material. This prolonged time was chosen to compensate for the intraoral and room temperature differences (81,93).

The retention screws were then removed from the impression copings and the custom tray was removed from the POM block. Implant replicas (ILA20 Implant Lab Analog 4.1 mm; Biomet 3i) were then fastened on the transfer impression copings by hand tightening the retention screws (54,75,78). The definitive impression was then boxed (Red Boxing Wax; Kemdent) and 60 min after the removal from the POM block it was poured with the gypsum product (51,77,94,95). Manufacturers’ instructions were followed for the mixing procedures. An electronic scale (EC-411; Acculab Sartorius Group) was used to accurately measure the gypsum powder’s weight, which was incorporated in distilled water (W5; Lidl Hellas) was previously measured and added in a vacuum bowl (Twister Venturi, Renfert). The dental stone was added and a laboratory spatula (3R; Buffalo Dental Mfg.) was used for a 15-second hand mixing to fully incorporate the powder into the water. Mechanical mixing under vacuum at 25 mm Hg for 45 seconds followed (68). The mixture was poured under vibration (Vibrator-P; Yamahachi Dental Mfg.) into the definitive impression. The definitive casts were left to set in the custom trays for 24 hours (50,68).

Three castable cylinders (GUCA2C UCLA Gold Non-Hexed Abutment Cylinder 4.1mm, Biomet 3i) with a machined non-enganging base were modified in order to create a circumferential 1.5 mm shoulder 5mm above the implants’ prosthetic platforms, using PMMA resin (Pattern Resin LS, GC America Inc). The total height of these cylinders was 8 mm. These cylinders were then cast in a high noble alloy (Mentor SF, Element Dental P). Three rings with an internal and an external diameter of 8mm and 5mm respectively, were connected with two plastic rods with a length of 20 mm and a diameter of 3.6 mm. This complex was then cast with a non-precious alloy (Rexillium III, Pentron). This metal structure had a passive fit on the 3 implant abutments, leaving a 0.6mm space between each ring and the corresponding abutment.

**Figure 3:**
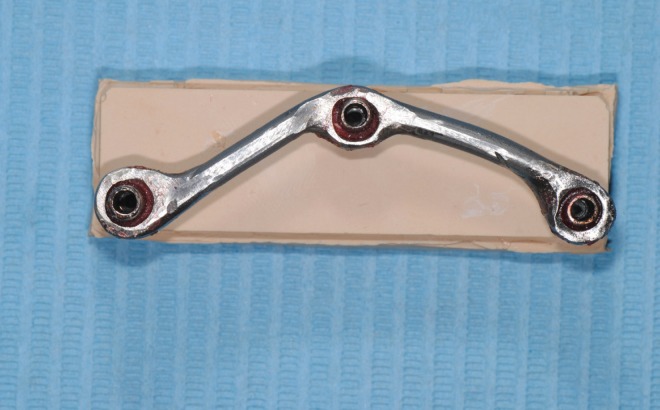
Cast implant-supported bar connected with PMMA resin.

**Figure 4:**
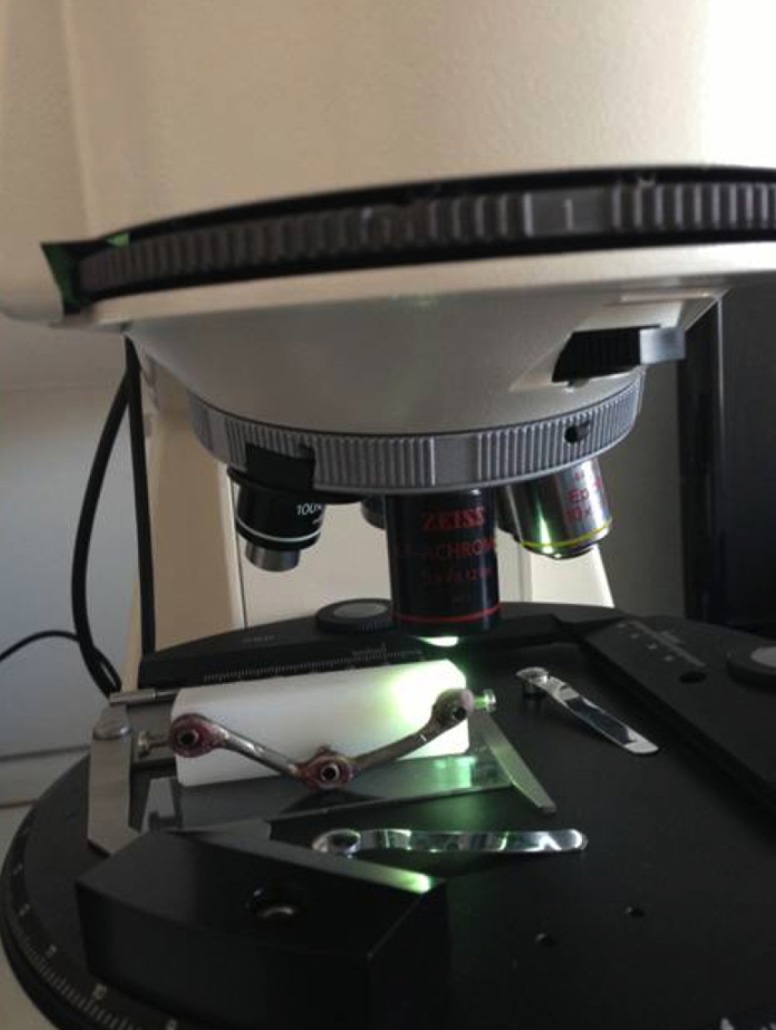
Optical microscope used for the measurements.

**Figure 5:**
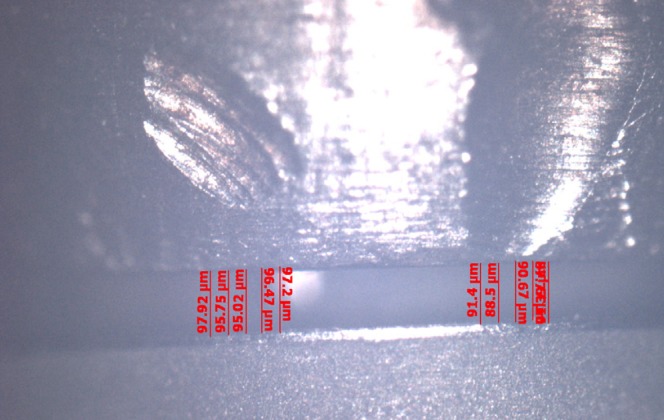
Marginal discrepancy as recorded by the special software used.

The abutments were tightened on the implant replicas and the metal framework was placed on the abutments and they were connected with PMMA resin (Pattern Resin LS, GC America Inc) using the bead brush technique, (Fig. 3). Twenty minutes after, the abutment screws were untightened and the abutment/framework complex was transferred to the POM block (83,97). Abutment screw no.1 was then tightened with a torque of 20N and the gap between the prosthetic platforms of implants no.2 and 3 and the corresponding abutments was measured using a special software (Axiovision, Carl Zeiss) and a camera (Axiocam ICc 1, Carl Zeiss) mounted on an optical microscope (Axioskop 40, Carl Zeiss) (29,98). A new screw was used every time. All measurements were made under a 10× magnification. The gap was measured by taking 10 measurements, 5 on each side of the center of the prosthetic platform (99). This method was employed since measurements could not be made towards the ends of the prosthetic platform due to its convexity and the blurriness of the image (80), (Figs. 4,5).

Twelve definitive casts were fabricated for each gypsum product. Forty measurements were performed for each cast: 10 measurements for each one of the abutments 1 and 3, and 20 for abutment 2. Therefore, 480 measurements were made for each dental stone, all by the same operator.

Room temperature (21±1oC) and relative humidity (50±10%) were recorded each day throughout the experiments, while a digital timer was used to standardize each procedure’s exact duration.

Mean, minimum, and maximum values were calculated. The Shapiro-Wilk test of normality was performed. Since the assumption of normality was not satisfied for all the measurements, Kruskal Wallis and Mann-Whitney non-parametric tests were used for the comparison of the different dental stones and the statistical analysis of the measurements. The coding used for the measurements is depicted in  Table 2.

**Table 2:**
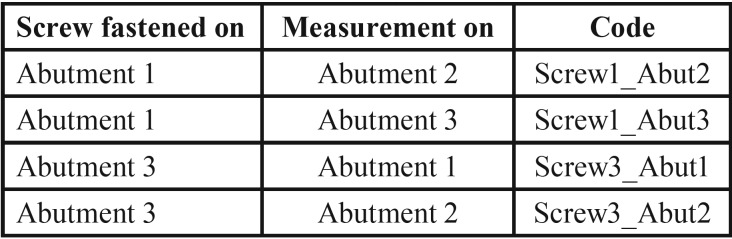
Coding used for the measurements.

## Results

Mean, minimum and maximum values (μm) for marginal discrepancies as related to various gypsum products and different screws fastened are summarized in  Table 3, Table 4.

**Table 3:**
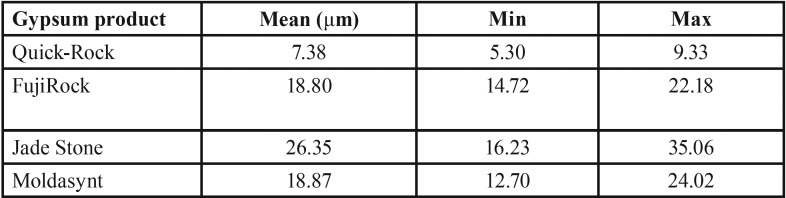
Mean, minimum, and maximum values (μm) for Screw1_Abut2 marginal discrepancies.

**Table 4:**
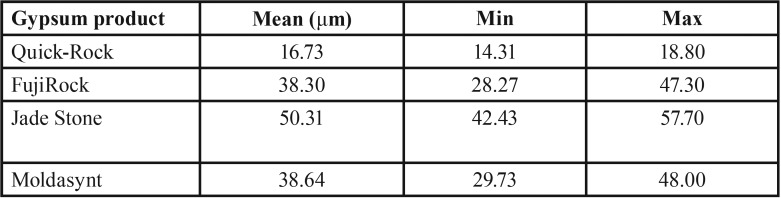
Mean, minimum, and maximum values (μm) for Screw1_Abut3 marginal discrepancies.

The non-parametric Kruskal-Wallis test revealed a statistically significant effect of the stone factor on the marginal discrepancy for all Sheffield test combinations: 1. Abutment 2 when screw was fastened on abutment 1 (χ2=3, df=35.33, *P*<0.01), 2. Abutment 3 when the screw was fastened on abutment 1 (χ2=3, df=37.74, *P*<0.01), 3. Abutment 1 when the screw was fastened on abutment 3 (χ2=3, df=39.79, *P*<0.01), 4. Abutment 2 when the screw was fastened on abutment 3 (χ2=3, df=37.26, *P*<0.01), ( Table 5, Table 6).

**Table 5:**
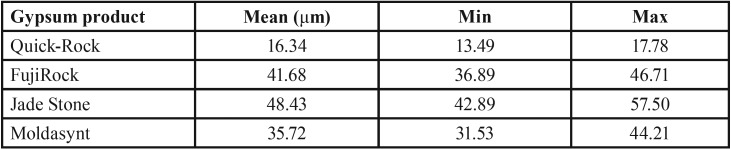
Mean, minimum and maximum values (μm) for Screw3_Abut1 marginal discrepancies.

**Table 6:**
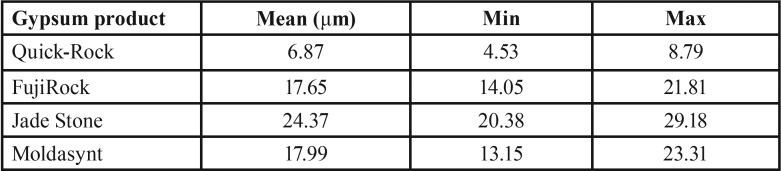
Mean, minimum, and maximum values (μm) for Screw3_Abut2 marginal discrepancies.

**Figure 6:**
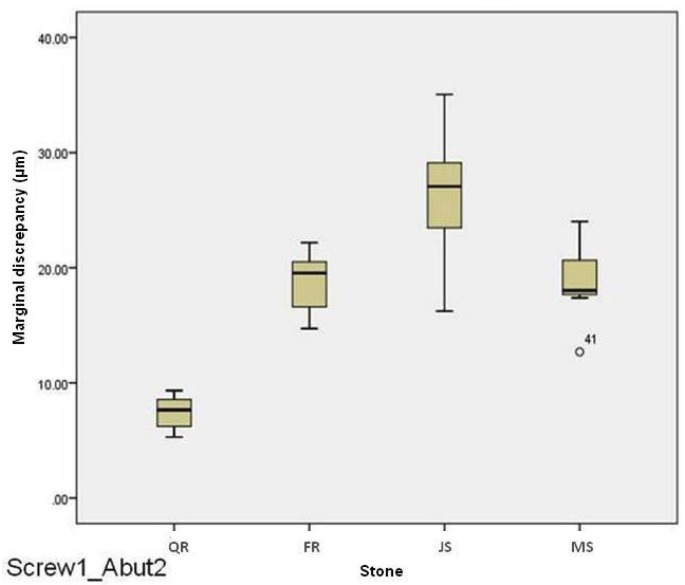
Box plots of the marginal discrepancy for abutment 2, when the screw was fastened on abutment 1.

For abutment 2, when the screw was fastened on abutment 1, the smallest marginal opening was noted for QuickRock (QR) with a mean value of 7.38μm, and the biggest was for Jade Stone (JS) with a mean value of 26.35μm ( Table 3). Box plots of marginal discrepancies for different gypsum products are shown in Fig. 6. The Mann-Whitney test (*P*<.06) was then used to test significant differences among the different stones, in sets of two ( Table 7, Table 8). Marginal discrepancies noted on abutment 2, when FujiRock (FR) and Moldasynt (MS) were used, were not statistically significant.

**Table 7:**
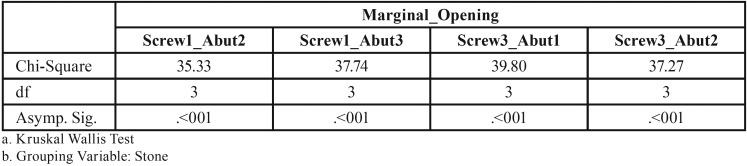
Kruskal-Wallis one-way analysis of variance.

**Table 8:**
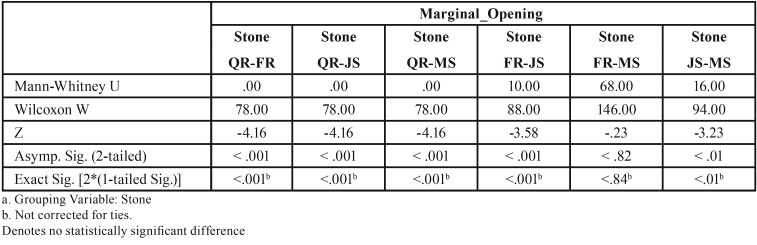
Mann-Whitney test (*P*<.05) for Screw1_Abut2 marginal discrepancies (μm) for different dental stones (N=12) (α=.05).

**Figure 7:**
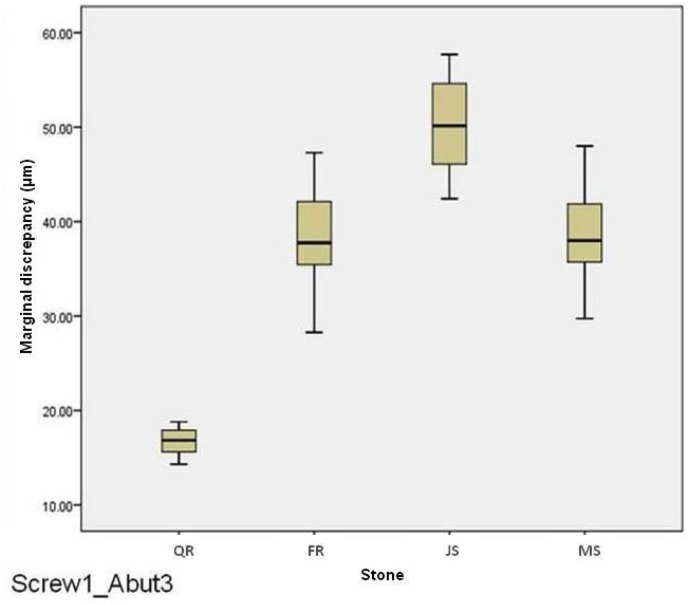
Box plots of the marginal discrepancy for abutment 3, when the screw was fastened on abutment 1.

For abutment 3, when the screw was fastened on abutment 1, the smallest marginal opening was noted for QuickRock with a mean value of 16.72μm, and the biggest was for Jade Stone with a mean value of 50.30μm ( Table 4). Box plots of the marginal discrepancy illustrating the means and the standard deviations for different gypsum products are shown in Fig. 7. Mann-Whitney test (*P*<.06) was then used to test significant differences among the different stones, in sets of two ( Table 9). Marginal discrepancies noted on abutment 2, when FujiRock (FR) and Moldasynt (MS) were used, were not statistically significant.

**Table 9:**
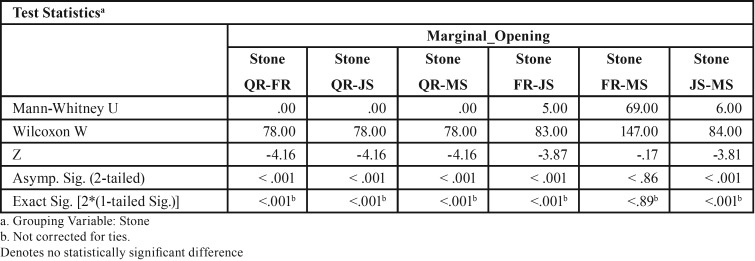
Mann-Whitney test for Screw1_Abut3 marginal discrepancies (μm) for different dental stones (N=12) (α=.05).

**Figure 8:**
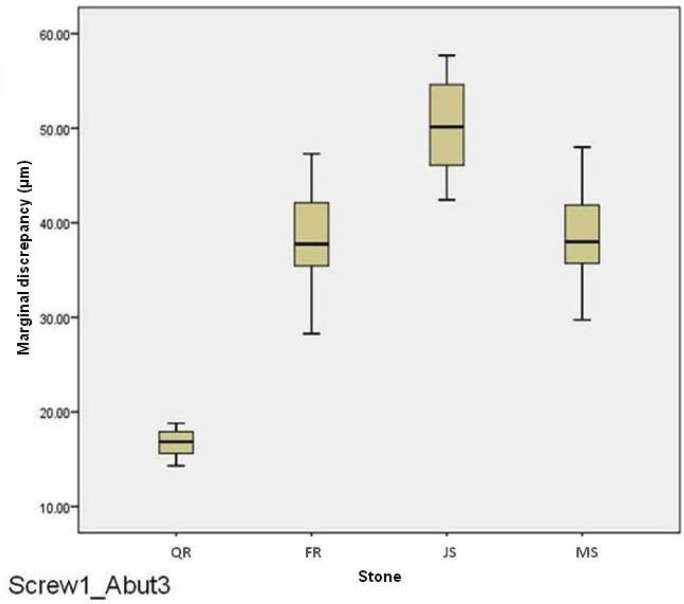
Box plots of the marginal discrepancy for abutment 1, when the screw was fastened on abutment 3.

For abutment 1, when the screw was fastened on abutment 3, the smallest marginal opening was noted for Quick Rock with a mean value of 16.34 μm, and the biggest was for Jade Stone with a mean value of 48.43μm ( Table 5). Box plots of the marginal discrepancy illustrating the means and the standard deviations for different gypsum products are shown in Fig. 8. The Mann-Whitney test (*P*<.06) was then used to test significant differences among the different stones, in sets of two ( Table 10). Marginal discrepancies noted on abutment 2 were statistically significant for all dental stones.

**Table 10:**
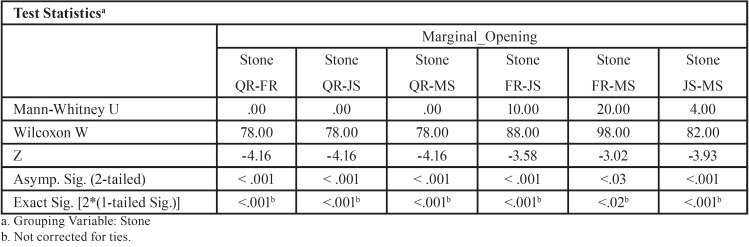
Mann-Whitney test for Screw3_Abut1 marginal discrepancies (μm) for different dental stones (N=12) (α=.05).

**Figure 9:**
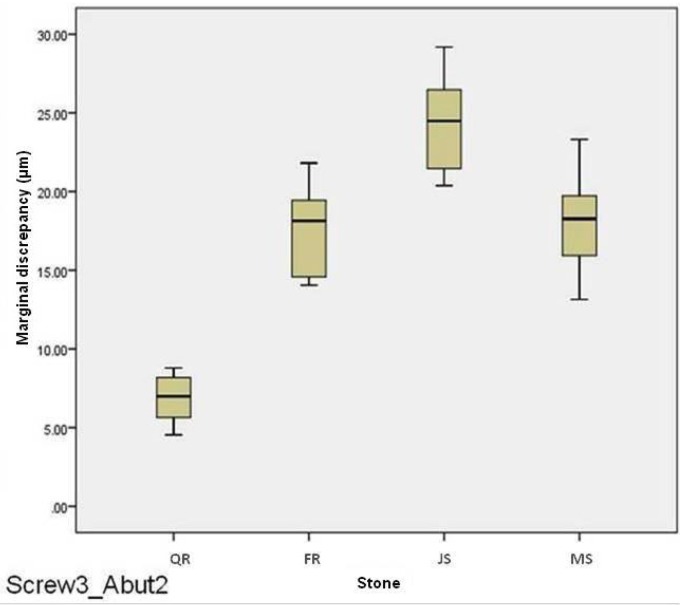
Box plots of the marginal discrepancy for abutment 2, when the screw was fastened on abutment 3.

For abutment 2, when the screw was fastened on abutment 3, the smallest marginal opening was noted for Quick Rock with a mean value of 6.87μm, and the biggest was for Jade Stone with a mean value of 24.37μm ( Table 6). Box plots of the marginal discrepancy illustrating the means and the standard deviations for different gypsum products are shown in Fig. 9. The Mann-Whitney test (*P*<.06) was then used to test significant differences among the different stones, in sets of two ( Table 11). Marginal discrepancies noted on abutment 2 - when FujiRock (FR) and Moldasynt (MS) were used - were not statistically significant.

**Table 11:**
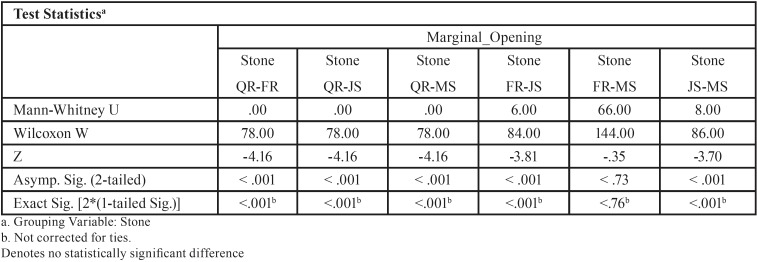
Mann-Whitney test for Screw3_Abut2 marginal discrepancies (μm) for different dental stones (N=12) (α=.05).

## Discussion

The study investigated the effect of dental stones on the accuracy of fitting of an implant supported screw-retained bar. The results of this study indicate that dental stones significantly affect the fitting of an implant supported screw-retained bar. Therefore, the null hypothesis was rejected.

Marginal adaptation between multiple abutments and implants’ prosthetic platforms is influenced by several factors including the impression material, impression techniques, dental stone used for the fabrication of the definitive models, wax properties, investment properties, investing procedures, alloy, casting and finishing procedures. The present study focused on only the gypsum products. Four commonly employed dental stones were used.

The results of the present study suggest that not all dental stones used for the fabrication of definitive casts provide an equally accurate fit of implant supported prostheses. It is evident that Quick Rock presents the best results, while Jade Stone the worst ones. Fujirock and Moldasynt, which are both type IV dental stones, did not differ significantly. These results were somewhat anticipated as Jade Stone is a type V dental stone, which is characterized as a high strength, high expansion stone.

The Sheffield test was adopted in this study, as it is widely clinically used (37,38). Moreover, the marginal discrepancy between the abutment and implant’s prosthetic platform was recorded and used as means of existence of passive fit or not. In that sense, none of the dental stones tested could provide a 100% passive fit, as marginal discrepancies ranged from 6.88μm to 50.31μm. The smaller marginal discrepancies were always noted at the middle implant. This is logical as the discrepancy is magnified as the distance becomes bigger (38).

As mentioned in the introduction, acceptable marginal discrepancies have ranged through the years, from 10 to 150 μm. In that manner, only Quick Rock was found to produce definitive casts which may provide fabrication of prostheses with marginal discrepancies close to the 10 μstrict standards that Brånemark, Romero *et al.* and Abduo and Lyons have set. This 10 μm standard could be achieved when the distance between two neighboring implants was 20 mm, but not when the distance was 56 mm. In that last case the marginal discrepancy was in the range of 16μm, which was less than the 26-30μm standard that Assif and Klineberg and Murray have set. Definitive casts made by type IV dental stones (FR and MS) produced marginal discrepancies which were smaller than the 26-30μm limit for the 20mm distance between the two implants, but not for the 56mm distance. The same applied for the type V dental stone. It should be mentioned however, that the prosthetic bars fabricated on the definitive casts produced by all gypsum products included in this study presented smaller marginal discrepancies than the 150μm limit set by Jemt in 1991 and Yanase in 1994 (10,37).

Marginal discrepancies below 20μm cannot be detected with either traditional x-ray films or digital periapical radiographs (49). Therefore, the 10μm acceptable marginal opening initially discussed by Brånemark cannot be perceived. Even, the 26-30μm acceptable marginal discrepancy limit falls very close to the detection capacity of the means available today.

The results of the present study agree with those of Wise (69), who found that definitive casts, on which implant supported fixed prostheses are fabricated, made by ultra-low expansion impression plaster are more accurate than casts made by conventional Type IV die stone. It should be mentioned however, that there are some differences both in the methodology and in the materials used between the study of Wise and the present study, with most profound the facts that Wise has used implant analogs instead of implants and fixed implant supported restorations made of impression plaster. On the contrary, in the present study a cast non-precious alloy framework connected to the abutments with a minimal amount of PMMA acrylic resin was employed. This was done in order to eliminate factors which could potentially influence the final outcome. These include wax distortion, investment expansion, alloy shrinkage and finishing procedures. Additionally, in the present study a mounting stone instead of an impression plaster has been used. It should be mentioned that Wise observed much bigger marginal discrepancies than the ones recorded in the present study. The use of casts instead of a rigid model to simulate the intraoral position of the implants, the expansion of the impression plaster indices which were used instead of a cast framework, the measurements that were made on projections of photographic slides and the use of laboratory analogs instead of implants may have contributed to the different numerical results.

The results of this study do not agree with those of Chang *et al.* (62) who concluded that the accuracy of the definitive casts is not influenced by the type of dental stone used. Nevertheless, there are some distinct differences between that study and the present one. First, Chang *et al.* investigated three impression techniques, two impression materials and two gypsum products, of which one was a type III and the other one a type IV. As already mentioned, the present study investigated only the influence of different gypsum products on the marginal discrepancy between the implant and the prosthesis. Second, the dimensional changes in that paper have been measured in the horizontal plane, while in the present one only the vertical discrepancy was measured.

Although every effort was made to standardize the multiple variables in the present study, some limitations were inevitably present. These included: a) the machining tolerance of the selected implant components (71-73), b) the fact that measurements were performed only in the vertical plane, c) the fact that mandibular flexure could not be taken into account, d) the temperature and dry environment where the impressions were made.

According to the results of the present study it may be preferable to use a mounting stone when fabricating prostheses supported by multiple implants, as this will decrease the marginal discrepancy between the implants’ prosthetic platforms and the corresponding abutments. However, if both implants and natural teeth have to be restored in the same arch, then the mounting stone cannot be used, since its Vickers hardness is 95MPa, and therefore a great risk of damage to the margins of the dies during the waxing procedures exists. In those cases a type IV dental stone should be employed. Another fact that should be taken into account is that type IV and type V dental stones provide sufficient working time, good detail reproduction and compatibility with elastomeric impression materials (60,61). On the other hand, mounting stones present a fast setting time, while their detail reproduction has not been investigated. Therefore the clinician has to weigh the advantages and disadvantages of using a mounting stone to fabricate a definitive cast.

Since no technique seems to be error-free, future studies should focus on comparing the use of dental stone products in combination with different impression materials and different implant components under simulated clinical conditions, taking also into account the operator variability. This methodology could provide valuable information and help the clinicians transfer laboratory findings to their everyday implant prosthodontics practice.

## Conclusions

Within the limitations of this *in vitro* study the following conclusions can be drawn:

1. A significant correlation exists between marginal discrepancy and different dental gypsum products used for the fabrication of definitive casts for implant supported bars.

2. The smallest marginal discrepancy was noted on implant supported bars fabricated on definitive casts made by Type III mounting stone.

3. The biggest marginal discrepancy was noted on implant supported bars fabricated on definitive casts made by Type V dental stone.

4. The marginal discrepancies presented on implant supported bars fabricated on definitive casts made by two types of Type IV dental stone were not significantly different.
