# Correction: Accuracy of fit of implant-supported bars fabricated on definitive casts made by different dental stones

**DOI:** 10.4317/jced.532743

**Published:** 2019-01-01

**Authors:** Ioannis Kioleoglou, Argirios Pissiotis, Konstantinos Michalakis

**Affiliations:** 1 DDS, MS, Former postgraduate resident, Dept. of Prosthodontics, School of Dentistry, Faculty of Health Sciences, Aristotle University, Thessaloniki, Greece. ITI Scholar and Honorary Clinical Research Fellow, Department of Restorative Dentistry and Adult Oral Health, Barts and the London School of Medicine and Dentistry, Queen Mary University of London, United Kingdom; 2 DDS, MS, PhD, Professor and Chair, Division of Removable Prosthodontics, Dept. of Prosthodontics, School of Dentistry, Faculty of Health Sciences, Aristotle University, Thessaloniki, Greece; 3 DDS, MSc, PhD, FACP, Associate Professor and Clinical Director of Postgraduate Prosthodontics, Dept. of Prosthodontics, School of Dentistry, Faculty of Health Sciences, Aristotle University, Thessaloniki, Greece. Adjunct Associate Professor, Division of Postgraduate Prosthodontics, Dept. of Prosthodontics, Tufts University School of Dental Medicine, Boston, MA, USA

In this article by Kioleoglou and colleagues ( *J Clin Exp Dent* 2018;3: 252-263 1 Mar), there is an error in the authors of the manuscript. The correct author list is: Ioannis Kioleoglou, Argirios Pissiotis, Konstantinos Michalakis.
